# Decreasing cloud cover drives the recent mass loss on the Greenland Ice Sheet

**DOI:** 10.1126/sciadv.1700584

**Published:** 2017-06-28

**Authors:** Stefan Hofer, Andrew J. Tedstone, Xavier Fettweis, Jonathan L. Bamber

**Affiliations:** 1 School of Geographical Sciences, University of Bristol, Bristol, UK.; 2 Laboratory of Climatology, Department of Geography, University of Liège, Liège, Belgium.

**Keywords:** Greenland, Greenland ice sheet, Cloud cover, Mass balance, albedo, Climate Change, Sea-level rise, Climatology, remote sensing, Climate modelling

## Abstract

The Greenland Ice Sheet (GrIS) has been losing mass at an accelerating rate since the mid-1990s. This has been due to both increased ice discharge into the ocean and melting at the surface, with the latter being the dominant contribution. This change in state has been attributed to rising temperatures and a decrease in surface albedo. We show, using satellite data and climate model output, that the abrupt reduction in surface mass balance since about 1995 can be attributed largely to a coincident trend of decreasing summer cloud cover enhancing the melt-albedo feedback. Satellite observations show that, from 1995 to 2009, summer cloud cover decreased by 0.9 ± 0.3% per year. Model output indicates that the GrIS summer melt increases by 27 ± 13 gigatons (Gt) per percent reduction in summer cloud cover, principally because of the impact of increased shortwave radiation over the low albedo ablation zone. The observed reduction in cloud cover is strongly correlated with a state shift in the North Atlantic Oscillation promoting anticyclonic conditions in summer and suggests that the enhanced surface mass loss from the GrIS is driven by synoptic-scale changes in Arctic-wide atmospheric circulation.

## Teaser

Reduction in summer cloud cover over the Greenland Ice Sheet is the main driver of recent melt.

## INTRODUCTION

The mass balance of the Greenland Ice Sheet (GrIS) has changed significantly over the last two decades. Until the mid-1990s, losses from surface meltwater runoff and ice discharge into the ocean (*D*) were roughly balanced by snow accumulation (*1*, *2*). However, since then, mass loss has accelerated (*3*) as the surface mass balance (SMB) has declined and *D* has increased (*1*), with a possible link between meltwater production and ice dynamics (*4*, *5*). As a consequence, the GrIS has become the dominant source of barystatic sea level rise, with an average (1991–2015) contribution of 0.47 ± 0.23 mm/year [equivalent to 171 gigatons (Gt) of ice] (*2*).

About 60% of this recent mass imbalance has been associated with a declining SMB predominantly due to enhanced surface melt (*1*, *2*). Studies based on in situ observations suggest that surface melt rates are controlled by variations in summertime shortwave (SW) radiation (*6*, *7*). However, to date, only the impact of a declining albedo (α) on the SW radiation budget (Eq. 1) has been considered (*8*, *9*).

$$\text{Net shortwave radiation}\;(\text{SWnet})={\text{SWD}}^{*}(1-\alpha )$$ (1)

It has previously been suggested that cloud cover has a positive feedback on melt rates by controlling longwave fluxes (*10*). Here, we use a combination of satellite cloud data and modeled radiation fluxes to assess the impact of recent changes in GrIS cloud cover upon radiative fluxes and, in turn, the SMB of the GrIS.

## RESULTS

### Trends in summer cloud cover

We use satellite-derived cloud products from (i) the Moderate Resolution Imaging Spectroradiometer (MODIS) sensor (*11*) on board NASA’s Aqua satellite and (ii) the Advanced Very High Resolution Radiometer (AVHRR) (*12*) to quantify cloud cover changes. We also use a regional climate model, Modèle Atmosphérique Régional (MAR), forced by the European Centre for Medium-Range Weather Forecasting (ECMWF) reanalysis (ERA), to assess the subsequent impact on SMB (see Materials and Methods) (*1*, *2*).

**Fig. 1:**
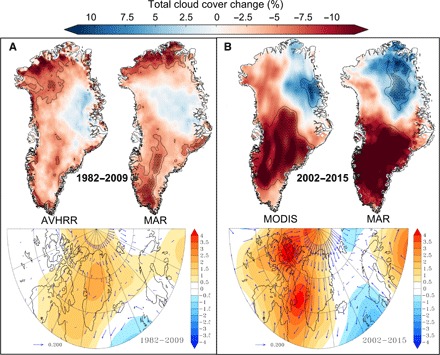
Total change in summer (JJA) cloud cover from satellites and a regional climate model. (**A**) Comparison between AVHRR (*12*) (left, top) and MAR (right, top) total JJA cloud cover change (%) during the full available data period of AVHRR between 1982 and 2009. Bottom: Trend of JJA 500-hPa geopotential height (Z500) in meters per year. The arrows show the wind trend in meters per second per year and highlight the circulation anomalies induced by the JJA Z500 changes. The arrow length of a change of 0.2 m/s per year is given in the legend for indication. (**B**) Comparison is the same as in (A) but for MODIS (*11*) (left; full observation period, 2002–2015) and MAR (right; 2002–2015). Values inside the black line have a significance level of *P* < 0.10, and the dotted areas indicate statistical significance at *P* < 0.05. All cloud cover trends are individually based on a linear regression analysis for every pixel.

Satellite observations reveal that there have been significant reductions in summertime [June-July-August (JJA)] optically thick cloud cover. Observations from AVHRR (Fig. 1A) recorded reductions in cloud cover of more than 84% of Greenland’s area over the time period 1982–2009. Over the same time period, MAR shows a reduction of more than 82%. During the period 2002–2015 (Fig. 1B), observations from MODIS show a cloud cover decrease of more than 77% of Greenland’s area, compared with 68% from MAR.

Outputs from MAR show good agreement in the spatial distribution and amplitude of the changes observed by both satellite platforms. The largest reductions are seen in the warmer west and south of Greenland, whereas cloud cover increased in the colder and drier northeast. The decrease in cloud cover that occurred after 2002 is relatively large, with substantial parts of southern Greenland experiencing a reduction of more than 10%. These cloudiness changes are a direct response to the circulation changes observed since the end of the 1990s (*13*). As shown in Fig. 1 (A and B, bottom), these circulation changes favor more anticyclonic conditions (warm and dry) over the south of Greenland except in the northeast where they favor southward fluxes (wet and cold), explaining the cloudiness increase in this area. During 1982–2009, the increases in geopotential height of the 500-hPa pressure level (Z500) promoted more anticyclonic conditions over most of Greenland, whereas during 2002–2015, the Z500 increases are limited to the west coast.

Although the spatial distribution of cloud cover is similar between MAR and the observations, the model slightly overestimates the area with cloud cover increase (32%) compared to the observations (23%). Nonetheless, the MAR trends and behavior are suitable for exploring the role that changing cloud cover has had on surface melt and, in turn, the SMB of the ice sheet.

**Fig. 2:**
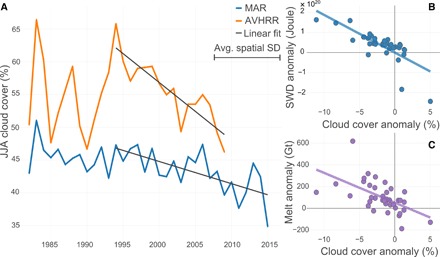
Summer (JJA) cloud cover time series, trends, and impacts of clouds on melt and radiation. (**A**) Time series of average Greenland cloud cover from AVHRR sensor (orange; 1982–2009) and MAR (blue; 1982–2015). Linear fit (dark gray): AVHRR (1994–2009): *R*^2^ = 0.76, *P* < 0.001; MAR (1994–2015): *R*^2^ = 0.46, *P* < 0.001. Length of one average spatial SD is shown in the legend. (**B**) Correlation between MAR JJA cloud cover anomalies and JJA SWD anomalies (*R*^2^ = 0.58, *P* < 0.001). A similar scatterplot showing the correlation between cloud cover and LWD anomalies can be found in fig. S3. (**C**) Correlation between JJA cloud cover anomalies and JJA melt anomalies (*R*^2^ = 0.32, *P* < 0.001). All anomalies in (B) and (C) are calculated on the basis of the 1970–1995 average.

Figure 2A (orange line) shows the average JJA cloud cover retrieved from AVHRR over Greenland from 1982 to 2009. It is characterized by two phases: From 1982 to ~1994, there is high interannual variability (SD, 6.1%) and no statistically significant trend. Around 1995, the behavior changes as a result of changes in general circulation reflected in a decreasing JJA North Atlantic Oscillation (NAO) index (*13*). The detrended interannual variability (SD, 2.3%) decreases by more than half, and the cloud cover shows a statistically significant negative trend between 1994 and 2009 (−0.9 ± 0.28% per year, *P* < 0.001, *R*^2^ = 0.76). The total change in cloud cover is −14.1%, which is larger than the average spatial SD (10.1%) and markedly above the interannual variability. This coincides with a shift in the NAO to an extremely negative state and with a shift in the Greenland Blocking Index (GBI) (*14*) to an extremely positive state (figs. S1 and S2), which appears anomalous for at least the last 160 years. These anomalies in both indexes suggest a higher frequency of anticyclonic conditions over Greenland, where the NAO index is susceptible to changes in mean surface pressure conditions over the North Atlantic (*13*), whereas the GBI is proportional to the general circulation directly over the GrIS at the 500-hPa level (*14*). Figure 2A (blue line) presents the average JJA cloud cover from MAR. It also shows a statistically significant trend from the mid-1990s to 2015, but less marked (−0.3 ± 0.17% per year), and the total change during the period is −6.1%, which is below the spatial variability (10.4%), but above the detrended interannual variability of 2.3%. The underestimation of cloud cover decline appears to be a consequence of the overestimation of the spatial extent of cloud cover increase (1982–2009, 2% of Greenland; 2002–2015, 9%) when comparing MAR to the observations (Fig. 1). These differences are likely due to small biases in temperature, humidity, and wind simulated by MAR and the nonlinear behavior of simulated cloud cover in regional climate models.

### Sources of increase in melt

**Fig. 3:**
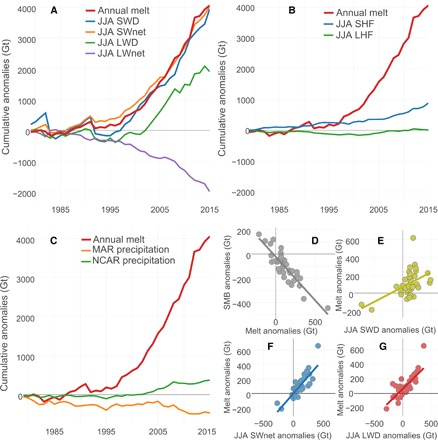
Accumulated melt anomalies and contributing factors. (**A**) Accumulated annual melt anomalies (in Gt) and JJA SWD, SWnet, LWD, and LWnet (net longwave radiation) anomalies. Radiation anomalies converted from joules to “melt potential” (in Gt) (see Materials and Methods). Anomalies are based on the 1970–1995 average of MAR (Eq. 2), and the accumulation of anomalies starts in 1979. (**B**) Description the same as in (A) but showing JJA latent heat flux (LHF) and sensible heat flux (SHF). (**C**) Accumulated annual precipitation anomalies (MAR and NCEP version 1 reanalysis) and annual melt anomalies. NCAR, National Center for Atmospheric Research. (**D**) Correlation between annual melt anomalies and annual SMB anomalies (*R*^2^ = 0.77, *P* < 0.001). (**E**) Correlation between JJA SWD anomalies and melt anomalies (*R*^2^ = 0.26, *P* < 0.001). (**F**) Correlation between JJA SWnet anomalies and melt anomalies (*R*^2^ = 0.75, *P* < 0.001). (**G**) Correlation between JJA LWD anomalies and melt anomalies (*R*^2^ = 0.63, *P* < 0.001).

On the basis of the correlation between JJA cloud cover and JJA shortwave downward (SWD) radiation anomalies (Fig. 2B), we find that for every percent of negative JJA cloud cover anomaly, Greenland receives 1.9 × 10^19^ ± 0.6 × 10^19^ J of extra SWD energy during summer melt season. We also find that for every percent of JJA cloud cover reduction, the melt during summer is enhanced by 27 ± 13 Gt (Fig. 2C). Next, we partitioned melt anomalies estimated by MAR into their different contributions (Fig. 3), converting radiative flux anomalies from joules into melt potential (in Gt), wherever applicable (see the “Experimental design” section).

Figure 3A shows the accumulated, annual MAR melt anomalies, with a strong positive trend (more melt) from about 1995, resulting in an accumulated melt anomaly of +3971 Gt between 1995 and 2015, which is in good agreement with other studies (*1*, *2*). We find a strong increase in both SWD (+4112) and SWnet (+3737 Gt) JJA anomalies during the same period. Whereas the increase in SWD is fully driven by the decrease in cloud cover during summer, the increase in SWnet is a result of both the increase in SWD (reduced cloud cover) and the coinciding decrease in surface albedo (*8*, *9*).

We also find that the increase in JJA longwave downward (LWD) radiation anomalies, which is directly proportional to the free atmospheric temperature in summer and global warming, has contributed less to the energy balance over the GrIS than SWD anomalies (+2277 Gt versus +4112 Gt). We also find that LWD anomalies are not sensitive to summer cloud cover anomalies (*R*^2^ = 0.003; fig. S3). The negative anomalies in LWnet radiation (−1669 Gt) indicate that the surface of the GrIS has recently been warming more than the atmosphere because of the combined effect of increased SW and longwave radiation reaching the surface.

Therefore, the exceptional melt of the GrIS since the mid-1990s has appeared to be a result of increases in both of the “external” drivers of the surface energy balance, LWD and SWD. Whereas previous studies have focused on the role of rising temperatures as the main cause of the current melt increase and albedo decline over the GrIS [for example, (*15*, *16*)], our results strongly indicate that it is rather a combination of increased SWD due to reduced cloud cover in summer combined with an increase in LWD due to higher free-atmosphere temperatures causing melt and surface darkening. Therefore, the decrease in surface albedo due to the melt-albedo feedback (*8*), which increases surface melt by increasing the ratio of absorbed solar radiation, has also been partly driven by a recent decrease in summer cloud cover enhancing the melt-albedo feedback (see also fig. S4) and not only by temperature anomalies.

Other studies have indicated that in the western ablation zone, nonradiative energy fluxes can play a significant role in enhancing short-term melt events (*17*). Although this can be the case for specific events on a small spatial and temporal scale, our results (Fig. 3B) indicate that over longer time periods and over the whole GrIS, sensible and latent heat flux have contributed very little extra energy to the surface energy increase of the GrIS (+630 and +119 Gt, respectively). To exclude the possibility that changes in precipitation patterns have contributed to the recent decline in SMB, we have analyzed two independent data sets of precipitation over Greenland (Fig. 3C). Whereas MAR driven by ERA shows a slightly negative precipitation trend (−143 Gt), National Centers for Environmental Prediction (NCEP) reanalysis indicates marginal precipitation increase between 1995 and 2015 (+446 Gt).

Our results also show that melt anomalies are the main factor in controlling total annual SMB anomalies (Fig. 3D). MAR indicates that 77% of the variability in SMB anomalies is controlled by melt anomalies, whereas melt anomalies are the main driver of meltwater runoff anomalies from the GrIS (*R*^2^ = 0.98; fig. S5). This agrees with the findings of van den Broeke *et al*. (*1*, *2*) who used gravimetry and climate model data to show that surface melt anomalies are the main driver of the recent mass loss from the GrIS. We also find that summertime SWD anomalies, due to a reduction in cloud cover, directly explain 26% of the variability in melt anomalies (Fig. 3E). JJA SWnet anomalies show an even stronger correlation with summertime melt anomalies, explaining 75% of the variability (Fig. 3F). In reality, parts of this strong correlation are also a manifestation of SWD anomalies, because under sunny conditions, the albedo of the GrIS is automatically lower than that under overcast conditions; this is due to clouds filtering out parts of the spectrum where the surface albedo is very low [near-infrared (*18*)]. This direct effect of clouds on the albedo is accounted for in MAR’s albedo scheme but does not show up in the correlation between melt and SWD anomalies. JJA LWD anomalies (Fig. 3G), although smaller in magnitude than SWD and SWnet, also show significant overlap with summer melt anomalies (*R*^2^ = 0.63). This effect is a direct consequence of Arctic free-atmosphere temperature increase. However, because the emissivity of the atmosphere, ε, is higher under cloudy conditions, the decrease in summertime cloud cover might have had some dampening effect on global warming and associated increase in LWD (the increase in LWD is lower in Fig. 3A during phases with high SWD and vice versa).

### Influence of large-scale circulation patterns

Fettweis *et al*. (*13*) reported on the importance of an anomalously low NAO index during summer and a subsequent increase in high-pressure frequency over Greenland. Synoptic-scale ridges in the mid-troposphere and high-pressure systems near the surface lead to large-scale sinking motion, which enhances cloud dissipation, leading to reduced cloud cover. The 5-year average of the NAO index has not been consistently negative since around 1960 (fig. S1) (*19*). The extended GBI time series (fig. S2) (*14*) indicates that it reached its highest values in the 2000s, a high that has not been seen since 1850 (>3 SD outside the mean).

**Fig. 4:**
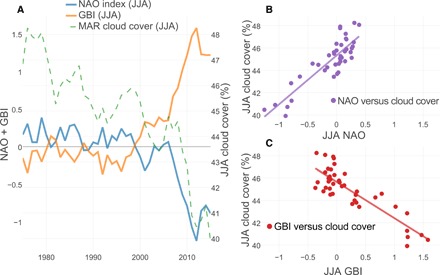
Correlation between cloud cover (model) and measured NAO/GBI index during summer (JJA). (**A**) Five-year running average of MAR JJA cloud cover (green; %), JJA NAO index (blue), and JJA GBI (orange). (**B**) The scatterplot (purple) shows the correlation (*R*^2^ = 0.75, *P* < 0.001) between observed NAO index and MAR cloud cover (both for JJA). (**C**) The scatterplot (red) shows the correlation (*R*^2^ = 0.74, *P* < 0.001) between GBI and MAR cloud cover (both for JJA).

We find that these changes in synoptic-scale circulation patterns and the associated increase in high-pressure frequency over Greenland (*13*) correlate strongly with changes in summertime cloud cover (*R*^2^ = 0.75, *P* < 0.001; Fig. 4, A and B). For every 0.2 decrease in JJA NAO index, gauging the general circulation at the surface over the North Atlantic, the JJA cloud cover has reduced by 0.88 ± 0.16%, with time periods of especially low cloud cover (such as 2010–2015) coinciding with a very negative NAO. There is also strong agreement between the JJA GBI (*14*), representative of the general circulation at 500 hPa over the GrIS, and JJA cloud cover (*R*^2^ = 0.74, *P* < 0.001; Fig. 4, A and C), confirming that the decrease of cloudiness over Greenland is due to synoptic-scale circulation changes and, in particular, due to the increase of anticyclonic conditions over Greenland, for which GBI is particularly sensitive. This strong correlation between summertime NAO index and the MAR-based cloud cover could be used to forecast whether the observed reduction in cloud cover during summer, and the associated increase in GrIS melt, is likely to continue. If it is linked to global warming and the poleward migration of large-scale circulation patterns (*20*), then global circulation models (GCMs) could be used to test this hypothesis. However, Franco *et al*. (*21*) have reported that GCM-forced regional climate models show an opposite trend in SW radiation compared to when they are driven by reanalysis data, because GCMs do not project change in general circulation over Greenland. It is interesting to note that only one of the Coupled Model Intercomparison Project Phase 5 GCMs simulates the recent extreme low in NAO index (*13*), and this extreme negative phase continues throughout the 21st century (*13*).

## DISCUSSION

Our results indicate that climate models that do not adequately capture NAO behavior will not reproduce the forcing required to simulate the current SMB trends observed over the last two decades, confirming the results of previous studies (*13*, *16*, *22*, *23*). They also indicate that the sudden decline in Greenland’s (surface) mass balance is not primarily a direct response to the local increase in atmospheric temperature, because anomalies in downwelling longwave radiation have contributed less energy to the increase in melt of the GrIS than SWD anomalies. This is contradictory to previous analyses that have focused on the increase in temperature as the main cause of GrIS melting (*15*, *16*), as well as on the longwave warming effect of clouds (*10*). Climate warming is instead altering large-scale circulation patterns (Fig. 4 and figs. S1 and S2) (*13*, *20*, *22*, *23*), which then causes an even larger response in the local energy budget of the GrIS by enhancing not only the atmospheric temperature but also the solar insolation. Furthermore, our results indicate that the recent decline in surface reflectivity is partly caused by SWD anomalies by enhancing the melt-albedo feedback and the spectrum of radiation reaching the surface (fig. S4) (*8*, *18*).

In addition, we note that it is essential that simulations used for future projections capture both the seasonal and spatial patterns of cloud cover changes, if they are to provide useful forcing to model future GrIS mass trends. This will be challenging given the relatively coarse resolution of the current generation of GCMs and the difficulty in optimizing cloud properties for both low/mid-latitude and polar climates. Our results present a paradigm shift for understanding the role of optically thick clouds on the SMB of the GrIS.

## MATERIALS AND METHODS

### Experimental design

We chose to base our analysis on the fact that for the observed melt-induced reduction of the SMB on the order of 10^3^ Gt since the mid-1990s (*1*, *2*), significant amounts of extra energy are required. Therefore, this deviation from the stable state before the mid-1990s has to be depictable as a large-scale deviation from the long-term mean in energy fluxes toward the ice surface or atmospheric variables (that is, precipitation). Because most of Greenland’s melt occurs during the three summer months (JJA) and studies based on in situ observations clearly show that variations in SW radiation dominate SMB variability (*6*, *7*), we tested the hypothesis that incoming SW radiation (that is, reduced cloud cover) is partly causing these changes in the net SW balance, along with the observed reduction in surface albedo (*8*, *9*), with a possible link between the two (*8*). Wherever possible, we therefore converted radiation and heat flux anomalies to a melt potential, using the heat of fusion to melt 1 kg of ice, *H*_f_ = 333.55 kJ/kg, to make a direct comparison with melt anomalies as much as possible. We acknowledge the fact that this is an oversimplification of the physical processes involved as some of the additional energy may heat up the snowpack rather than directly influencing melt (and therefore, SMB). However, if parts of our presented energy surplus are used for processes such as heating the snowpack, then the correlation between melt potential (radiation and heat flux anomalies) and melt anomalies in our analysis will decrease, and therefore, our results represent a cautious estimate of the actual contribution to Greenland’s melting signal.

### Modèle Atmosphérique Régional

The MAR used in this study is a (non)hydrostatic regional climate model that solves the atmospheric primitive equation set [refer to the studies of Gallée and Schayes (*24*) and Fettweis (*25*) for a detailed description of the model]. MAR version 3.5.2 was used here (*26*). It was forced at its lateral boundaries every 6 hours by the ECMWF ERA-Interim (1979–2015) and ERA-40 data set (before 1979), with the atmospheric forcing fields containing temperature, wind, humidity, and surface pressure. Sea ice cover and sea surface temperature were also prescribed every 6 hours. Therefore, in terms of cloud cover trends, it was independent of the (cloud) data assimilation of MODIS and AVHRR data into the ERA fields. MAR was coupled with the multilayered one-dimensional energy balance–based snow model SISVAT (Soil Ice Snow Vegetation Atmosphere Transfer), which is used for the connection between atmosphere, snowpack, sea, and permanent ice as well as the snow-covered tundra [for a detailed description of SISVAT, see the study of De Ridder and Gallée (*27*)]. The snow-ice part in SISVAT was based on the snow model CROCUS (*27*, *28*). The cloud scheme of MAR was based on Meyers *et al*. (*29*) and on in situ measurements and subsequent model development during the Mixed-Phase Arctic Cloud Experiment (*30*) and was specially developed to depict Arctic cloud characteristics. The model setup and output of MAR were rigorously tested over the GrIS and tuned to match observed atmospheric and surface properties as closely as possible to depict trends in the SMB of the GrIS (*25*, *31*). It was validated against other regional climate models (*31*), and it also showed good agreement with in situ automatic weather station data and passive microwave remote-sensing data (*26*, *30*, *31*). The model was run on an equal-area 25 × 25–km grid, whereas the temporal coverage of the data in this study spanned from 1979 to 2015, except for the climatological base state (1970–1995).

### Computation of anomalies

The computation of anomalies from MAR (cloud cover, SMB, SW, longwave radiation components, etc.) was based on the 1970–1995 mean state of the model. If radiation and heat flux anomalies were not presented in the corresponding SI unit (joule), then we used the heat of fusion to melt 1 kg of ice, *H*_f_ = 333.55 kJ/kg, to convert the radiation anomaly in a corresponding mass anomaly (melt potential). A positive melt anomaly corresponds to an above-average downward flux of radiation and vice versa.

For every pixel of the 25 × 25–km grid, first, a monthly arithmetic mean from the daily model output was computed for every grid cell. Then, a climatology was produced, on the basis of an arithmetic mean of the period 1970–1995 for the specific month (January-December), and then, the deviation for every grid cell and month was computed as

$$a_{i,j,month}=x_{i,j,month}-\frac{\sum_{n=1970}^{1995}x_{ij,m,n}}{26}$$ (2)

where *a*_*i*,*j*,month_ is the deviation of the monthly values *x*_*i*,*j*,month_ from the specific monthly grid cell climatology (second term in Eq. 2), subscript *i*,*j* refers to the *i*th row and *j*th column of the model grid, and subscript *m* represents a fixed month for which the climatology is computed. If values of SMB anomalies were presented, we summed up (spatially) all the per-pixel deviations from the 1970–1995 mean state (*a*_*i*,*j*,month_) over the GrIS model domain to obtain one value for the specified time for the entire GrIS.

$$\Delta a_{total}=\sum_{i}\sum_{j}a_{i,j,month}$$ (3)

If radiation anomalies were presented, we first calculated the per-pixel deviations as given by Eq. 2 to obtain the deviation from the 1970–1995 mean in watts per meter square. Because every grid cell in the model configuration had a spatial coverage of 25 × 25 km, we multiplied this value by the grid-cell area to obtain the total deviation in watts (joules per second) per one grid cell. In the last step, we summed up all these values spatially (as in Eq. 3) and multiplied it by the time of the month (in seconds) to get from joules per second to total deviation in joules.

### MODIS cloud cover

The MODIS has been operational since 2000 on board two satellites, Aqua and Terra. It has a cross-track swath width of 2330 km and an along-track swath length of 10 km. It makes use of measurements at 36 different wavelengths, ranging from 0.4 to 14.4 μm, of which 14 are used to test whether clouds are present (*32*, *33*). Most of the tests are designed to identify contrast between clouds and the atmosphere or the surface.

Here, we used the global, monthly fractional cloud cover product (MYD08_M3) (*11*) from the MODIS sensor on board of the polar orbiting Aqua satellite, which was computed by summarizing the daily level 3 product over one calendar month (*34*). Cloud fraction within this data set was defined as the ratio between the sum of cloudy pixels and the total number of pixels within one grid cell. The measurement period available for this study ranged from 2002 to 2015. The spatial grid of the monthly cloud cover product was an equal-angle 1° longitude by 1° latitude grid, with the daily level 2 product (MOD06_L2) underlying the daily level 3 product that had a higher spatial resolution of 1 km (2, 5, and the remaining 29 bands were measured at resolutions of 250 m, 500 m, and 1 km, respectively) (*34*). For every 1° by 1° grid cell, all the underlying level 2 pixels were used to determine the cloud fraction over a period of 1 day and then were averaged over 1 month (*34*). For the computation of cloud cover averages over the entire GrIS, data points were weighted on the basis of their latitude to take into account the meridian convergence toward the North Pole.

### AVHRR cloud cover

The AVHRR is a broadband, four- to six-channel (depending on the sensor version) radiometer. The sensors have mostly been carried on different National Oceanic and Atmospheric Administration (NOAA) polar-orbiting satellites all forming a part of the Polar-Orbiting Environmental Satellites. The monthly cloud cover retrievals were provided by EUMETSAT (European Organisation for the Exploitation of Meteorological Satellites)’s Satellite Application Facility on Climate Monitoring (CM SAF). The CM SAF Global Area Coverage (GAC) Edition 1 data set provides an intercalibrated Climate Data Record (CDR) on fractional cloud cover based on AVHRR measurements, with the cloud characteristics (cloud fraction, cloud optical thickness, etc.) being produced with the Polar Platform System and the Cloud Physical Properties algorithms (*35*–*37*).

The information of the data set used in this study was provided on a 0.25° by 0.25° equal-angle latitude-longitude grid, with the underlying level 2 GAC data having a spatial resolution of 4 km (*12*). The temporal range of the available cloud fraction CDR was from 1982 to 2009. The data set was mostly verified by cross-checking with data from weather stations (surface synoptic observation reports) but was also validated against MODIS observations, with the targeted bias-corrected root mean square error lying between 15 and 20% (*37*). Newer AVHRR data were also available for 2009–2016, but they have not been processed to reach the status of an intercalibrated CDR yet. As in the previous subsection, if area averages from AVHRR were presented, data points were weighted on the basis of their latitude.

### Comparison between MODIS and AVHRR

The main advantage of using MODIS and AVHRR data compared to earlier derived cloud climatologies is the wide spectral coverage and their relatively high spatial resolution (*38*). The International Satellite Cloud Climatology Project, for example, only used two spectral bands to detect clouds, one in the visible and one in the infrared, whereas the AVHRR cloud mask algorithm used six different bands. The higher resolution and number of spectral bands used in the MODIS product resulted in a more robust, but shorter-length, product than AVHRR. The detection limit for MODIS was found to be situated at a cloud optical thickness of τ ~ 0.4 (*39*). The validation of MODIS cloud cover with the Cloud-Aerosol Lidar with Orthogonal Polarization (CALIOP) aboard the Calipso satellite, with a detection limit of τ ~ 0.1, showed an overlap in 87% of cloud conditions (*39*). The difference was mainly due to the superior detection capability by CALIOP of clouds with a cloud optical thickness between τ ~ 0.4 and τ ~ 0.1.

Although the AVHRR cloud cover data set is less capable than MODIS, particularly in the detection of very thin clouds (and showing less total cloud amount), it is still capable of reproducing multiannual cloud cover trends for all the other types of clouds. Its biggest advantage is its longer time range. The first measurement in the data set was taken in 1982 (compared to 2002 with MODIS Aqua).

## Supplementary Material

http://advances.sciencemag.org/cgi/content/full/3/6/e1700584/DC1

## SUPPLEMENTARY MATERIALS

Supplementary material for this article is available at http://advances.sciencemag.org/cgi/content/full/3/6/e1700584/DC1 fig. S1. Long-term NAO index from observations on Iceland and the Azores (1950–2016) (*19*). fig. S2. Extended GBI (1850–2016) (*14*). fig. S3. Correlation between JJA cloud cover and LWD anomalies. fig. S4. Correlation between summer radiation anomalies and albedo. fig. S5. Correlation between annual melt and runoff anomalies.

## References

[1] van den Broeke M. R., Bamber J., Ettema J., Rignot E., Schrama E., van de Berg W. J., van Meijgaard E., Velicogna I., Wouters B., Partitioning recent Greenland mass loss. Science 326, 984–986 (2009). 19965509 10.1126/science.1178176

[2] van den Broeke M. R., Enderlin E. M., Howat I. M., Munneke P. K., Noël B. P. Y., van de Berg W. J., van Meijgaard E., Wouters B., On the recent contribution of the Greenland Ice Sheet to sea level change. The Cryosphere 10, 1933–1946 (2016).

[3] Wouters B., Bamber J. L., van den Broeke M. R., Lenaerts J. T. M., Sasgen I., Limits in detecting acceleration of ice sheet mass loss due to climate variability. Nat. Geosci. 6, 613–616 (2013).

[4] Rignot E., Box J. E., Burgess E., Hanna E., Mass balance of the Greenland Ice Sheet from 1958 to 2007. Geophys. Res. Lett. 35, L20502 (2008).

[5] Bamber J., van den Broeke M. R., Ettema J., Lenaerts J., Rignot E., Recent large increases in freshwater fluxes from Greenland into the North Atlantic. Geophys. Res. Lett. 39, L19501 (2012).

[6] van den Broeke M. R., Smeets C. J. P. P., van de Wal R. S. W., The seasonal cycle and interannual variability of surface energy balance and melt in the ablation zone of the west Greenland Ice Sheet. The Cryosphere 5, 377–390 (2011).

[7] van de Wal R. S. W., Greuell W., van den Broeke M. R., Reijmer C. H., Oerlemans J., Surface mass-balance observations and automatic weather station data along a transect near Kangerlussuaq, West Greenland. Ann. Glaciol. 42, 311–316 (2005).

[8] Box J. E., Fettweis X., Stroeve J. C., Tedesco M., Hall D. K., Steffen K., Greenland ice sheet albedo feedback: Thermodynamics and atmospheric drivers. The Cryosphere 6, 821–839 (2012).

[9] Dumont M., Brun E., Picard G., Michou M., Libois Q., Petit J.-R., Geyer M., Morin S., Josse B., Contribution of light-absorbing impurities in snow to Greenland’s darkening since 2009. Nat. Geosci. 7, 509–512 (2014).

[10] Van Tricht K., Lhermitte S., Lenaerts J. T. M., Gorodetskaya I. V., L’Ecuyer T. S., Noël B., van den Broeke M. R., Turner D. D., van Lipzig N. P. M., Clouds enhance Greenland Ice Sheet meltwater runoff. Nat. Commun. 7, 10266 (2016). 26756470 10.1038/ncomms10266 PMC4729937

[11] S. Platnick, MODIS Atmosphere L3 Monthly Product (NASA, 2015); http://dx.doi.org/10.5067/MODIS/MYD08_M3.006

[12] K. G. Karlsson, M. Stengel, CM SAF Cloud, Albedo, Radiation dataset, AVHRR-based, Edition 1 (CLARA-A1), Cloud Products. (1.1) (2014); http://dx.doi.org/10.5676/EUM_SAF_CM/CLARA_AVHRR/V001.

[13] Fettweis X., Hanna E., Lang C., Belleflamme A., Erpicum M., Gallée H., Important role of the mid-tropospheric atmospheric circulation in the recent surface melt increase over the Greenland Ice Sheet. The Cryosphere 7, 241–248 (2013).

[14] Hanna E., Cropper T. E., Hall R. J., Cappelen J., Greenland Blocking Index 1851–2015: A regional climate change signal. Int. J. Climatol. 36, 4847–4861 (2016).

[15] Tedesco M., Doherty S., Fettweis X., Alexander P., Jeyaratnam J., Stroeve J., The darkening of the Greenland Ice Sheet: Trends, drivers, and projections (1981–2100). The Cryosphere 10, 477–496 (2016).

[16] Tedesco M., Fettweis X., Mote T., Wahr J., Alexander P., Box J. E., Wouters B., Evidence and analysis of 2012 Greenland records from spaceborne observations, a regional climate model and reanalysis data. The Cryosphere 7, 615–630 (2013).

[17] Fausto R. S., Box J. E., Colgan W., Langen P. L., Mottram R. H., The implication of non-radiative energy fluxes dominating Greenland Ice Sheet exceptional ablation area surface melt in 2012. Geophys. Res. Lett. 43, 2649–2658 (2016).

[18] Warren S. G., Optical properties of snow. Rev. Geophys. 20, 67–89 (1982).

[19] Barnston A. G., Livezey R. E., Classification, seasonality and persistence of low-frequency atmospheric circulation patterns. Mon. Weather Rev. 115, 1083–1126 (1987).

[20] Norris J. R., Allen R. J., Amato E. T., Zelinka M. D., O’Dell C. W., Klein S. A., Evidence for climate change in the satellite cloud record. Nature 536, 72–75 (2016). 27398619 10.1038/nature18273

[21] Franco B., Fettweis X., Erpicum M., Future projections of the Greenland Ice Sheet energy balance driving the surface melt. The Cryosphere 7, 1–18 (2013).

[22] Hanna E., Fettweis X., Mernild S. H., Cappelen J., Ribergaard M. H., Shuman C. A., Steffen K., Wood L., Mote T. L., Atmospheric and oceanic climate forcing of the exceptional Greenland Ice Sheet surface melt in summer 2012. Int. J. Climatol. 34, 1022–1037 (2014).

[23] Hanna E., Cropper T. E., Jones P. D., Scaife A. A., Allan R., Recent seasonal asymmetric changes in the NAO (a marked summer decline and increased winter variability) and associated changes in the AO and Greenland Blocking Index. Int. J. Climatol. 35, 2540–2554 (2015).

[24] Gallée H., Schayes G., Development of a three-dimensional meso-γ primitive equation model: Katabatic winds simulation in the area of Terra Nova Bay, Antarctica. Mon. Weather Rev. 122, 671–685 (1994).

[25] Fettweis X., Reconstruction of the 1979–2006 Greenland Ice Sheet surface mass balance using the regional climate model MAR. The Cryosphere 1, 21–40 (2007).

[26] Fettweis X., Box J. E., Agosta C., Amory C., Kittel C., Lang C., van As D., Machguth H., Gallée H., Reconstructions of the 1900–2015 Greenland Ice Sheet surface mass balance using the regional climate MAR model. The Cryosphere 11, 1015–1033 (2017).

[27] De Ridder K., Gallée H., Land surface–induced regional climate change in southern Israel. J. Appl. Meteorol. 37, 1470–1485 (1998).

[28] Gallée H., Guyomarc’h G., Brun E., Impact of snow drift on the Antarctic ice sheet surface mass balance: Possible sensitivity to snow-surface properties. Bound.-Lay. Meteorol. 99, 1–19 (2001).

[29] Meyers M. P., DeMott P. J., Cotton W. R., New primary ice-nucleation parameterizations in an explicit cloud model. J. Appl. Meteorol. 31, 708–721 (1992).

[30] Fridlind A. M., Ackerman A. S., McFarquhar G., Zhang G., Poellot M. R., DeMott P. J., Prenni A. J., Heymsfield A. J., Ice properties of single-layer stratocumulus during the Mixed-Phase Arctic Cloud Experiment: 2. Model results. J. Geophys. Res. 112, D24202 (2007).

[31] Fettweis X., Tedesco M., van den Broeke M. R., Ettema J., Melting trends over the Greenland Ice Sheet (1958–2009) from spaceborne microwave data and regional climate models. The Cryosphere 5, 359–375 (2011).

[32] Ackerman S. A., Strabala K. I., Menzel W. P., Frey R. A., Moeller C. C., Gumley L. E., Discriminating clear sky from clouds with MODIS. J. Geophys. Res. 103, 32–141 (1998).

[33] Frey R. A., Ackerman S. A., Liu Y., Strabala K. I., Zhang H., Key J. R., Wang X., Cloud detection with MODIS. Part I: Improvements in the MODIS cloud mask for collection 5. J. Atmos. Ocean. Technol. 25, 1057–1072 (2008).

[34] P. Hubanks, S. Platnick, M. King, B. Ridgway, “MODIS Atmosphere L3 gridded product algorithm theoretical basis document (atbd) & users guide” (Collection 006, NASA, 2016); https://modis-atmos.gsfc.nasa.gov/_docs/L3_ATBD_C6.pdf.

[35] Dybbroe A., Karlsson K.-G., Thoss A., NWCSAF AVHRR cloud detection and analysis using dynamic thresholds and radiative transfer modeling. Part I: Algorithm description. J. Appl. Meteorol. 44, 39–54 (2005).

[36] Dybbroe A., Karlsson K.-G., Thoss A., NWCSAF AVHRR cloud detection and analysis using dynamic thresholds and radiative transfer modeling. Part II: Tuning and validation. J. Appl. Meteorol. 44, 55–71 (2005).

[37] K.-G. Karlsson, A. Riihelä, R. Müller, J. F. Meirink, J. Sedlar, M. Stengel, M. Lockhoff, J. Trentmann, F. Kaspar, R. Hollmann, E. Wolters, CLARA-A1: CM SAF clouds, Albedo and radiation dataset from AVHRR data - Edition 1 - Monthly means/daily means/pentad means/monthly histograms” (Satellite Application Facility on Climate Monitoring, 2012); http://dx.doi.org/10.5676/EUM_SAF_CM/CLARA_AVHRR/V001.

[38] King M. D., Platnick S., Menzel W. P., Ackerman S. A., Hubanks P. A., Spatial and temporal distribution of clouds observed by MODIS onboard the Terra and Aqua satellites. IEEE Trans. Geosci. Remote Sens. 51, 3826–3852 (2013).

[39] Holz R. E., Ackerman S. A., Nagle F. W., Frey R., Dutcher S., Kuehn R. E., Vaughan M. A., Baum B., Global Moderate Resolution Imaging Spectroradiometer (MODIS) cloud detection and height evaluation using CALIOP. J. Geophys. Res. 113 D00A19 (2008).

