# Supplementary material for: Decreasing cloud cover drives the recent mass loss on the Greenland Ice Sheet
Source: Sci Adv. 2017 Jun 28;3(6):e1700584. doi: 10.1126/sciadv.1700584 (PMC5489271; doi:10.1126/sciadv.1700584)
Supplement: http://advances.sciencemag.org/cgi/content/full/3/6/e1700584/DC1 [file supp_3_6_e1700584__index.html]

Science Advances | Science Advances

## Supplementary Materials

**This PDF file includes:**

- fig. S1. Long-term NAO index from observations on Iceland and the Azores (1950–2016) (*19*).
- fig. S2. Extended GBI (1850–2016) (*14*).
- fig. S3. Correlation between JJA cloud cover and LWD anomalies.
- fig. S4. Correlation between summer radiation anomalies and albedo.
- fig. S5. Correlation between annual melt and runoff anomalies.

Download PDF

**Files in this Data Supplement:**

- Adobe PDF - 1700584\_SM.pdf
