# Supplementary material for: Decreasing cloud cover drives the recent mass loss on the Greenland Ice Sheet
Source: Sci Adv. 2017 Jun 28;3(6):e1700584. doi: 10.1126/sciadv.1700584 (PMC5489271; doi:10.1126/sciadv.1700584)
Supplement: http://advances.sciencemag.org/cgi/content/full/3/6/e1700584/DC1 [file 1700584_SM.pdf]

Science Advances

AAAS

advances.sciencemag.org/cgi/content/full/3/6/e1700584/DC1

Supplementary Materials for

Decreasing cloud cover drives the recent mass loss on the Greenland Ice Sheet

Stefan Hofer, Andrew J. Tedstone, Xavier Fettweis, Jonathan L. Bamber

Published 28 June 2017, *Sci. Adv.* **3**, e1700584 (2017)
DOI: 10.1126/sciadv.1700584

This PDF file includes:

- fig. S1. Long-term NAO index from observations on Iceland and the Azores (1950–2016) (*19*).
- fig. S2. Extended GBI (1850–2016) (*14*).
- fig. S3. Correlation between JJA cloud cover and LWD anomalies.
- fig. S4. Correlation between summer radiation anomalies and albedo.
- fig. S5. Correlation between annual melt and runoff anomalies.

Supplementary Materials

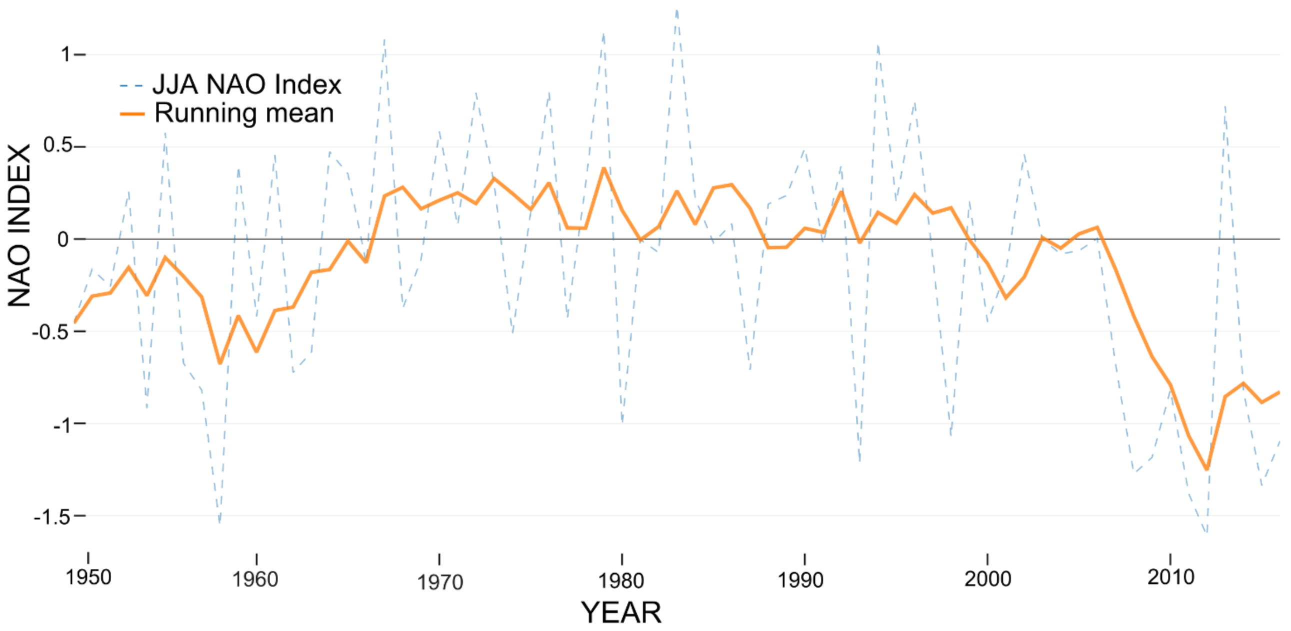

fig. S1. Long-term NAO index from observations on Iceland and the Azores (1950–2016) (*19*). Average JJA NAO index (blue) and 5 year running average (orange) retrieved from observations from the Azores (Ponta Delgada) and Iceland (Reykjavik).

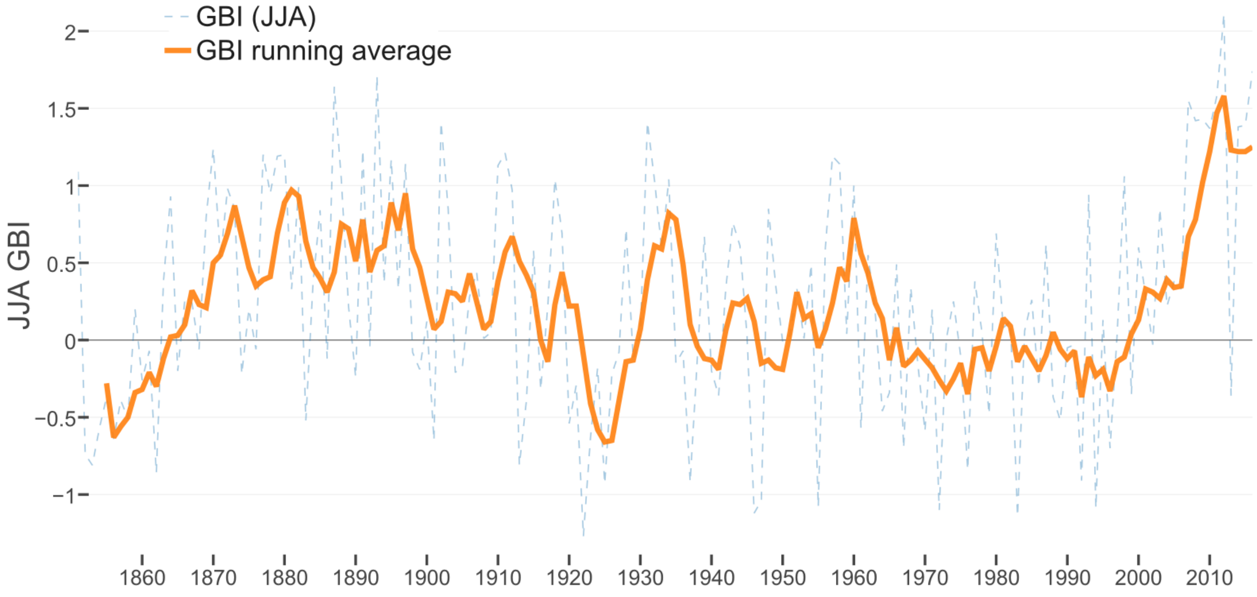

fig. S2. Extended GBI (1850–2016) (*14*). Average JJA GBI from mean 500 hPa geopotential height between 60-80°N and 20-80°W (blue) and 5 year running average GBI (orange).

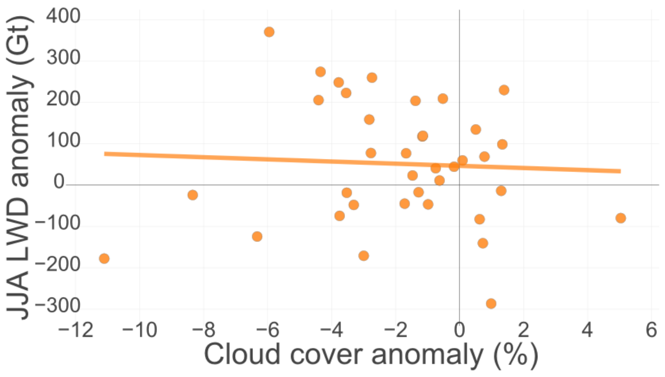

fig. S3. Correlation between JJA cloud cover and LWD anomalies. Scatterplot and linear regression line of JJA cloud cover anomalies (%) and JJA longwave downward anomalies (Gt) ($R^2 = 0.003$, $p = 0.1$).

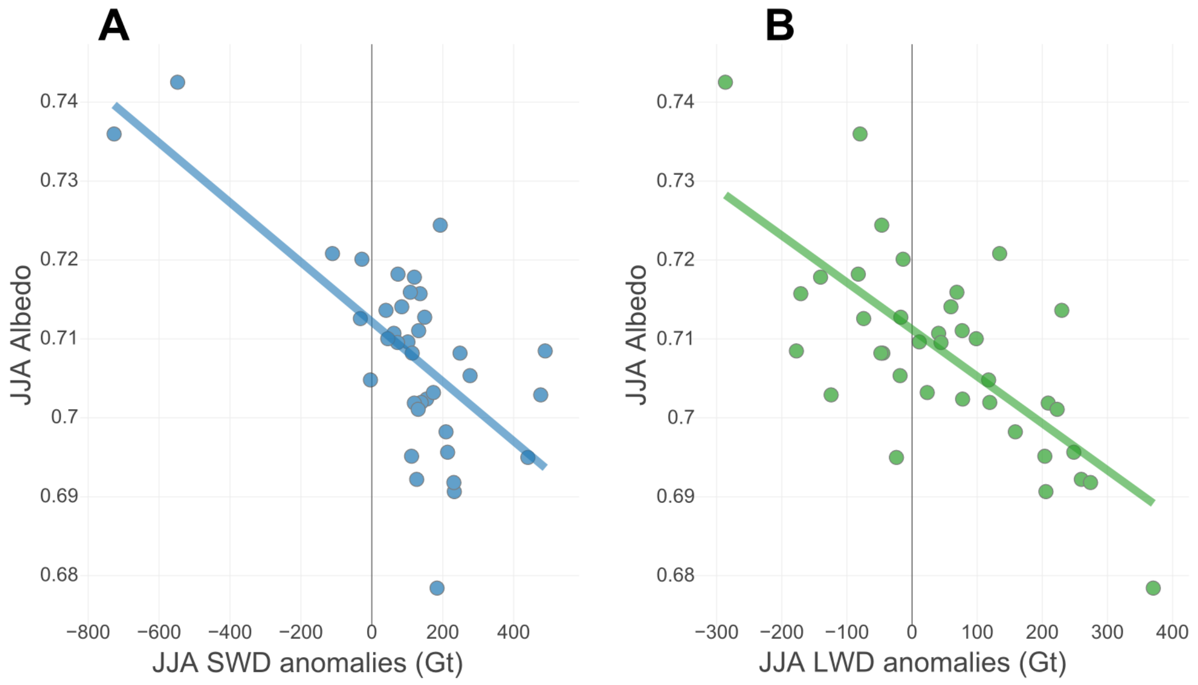

fig. S4. Correlation between summer radiation anomalies and albedo. (**A**), correlation between JJA SWD anomalies since 1979 and GrIS summertime albedo ($R^2 = 0.46$, $p < 0.001$), based on the 1970-1995 climatological mean of MAR, (**B**), same as **A**) but showing JJA LWD anomalies and summertime albedo correlation ($R^2 = 0.50$, $p < 0.001$).

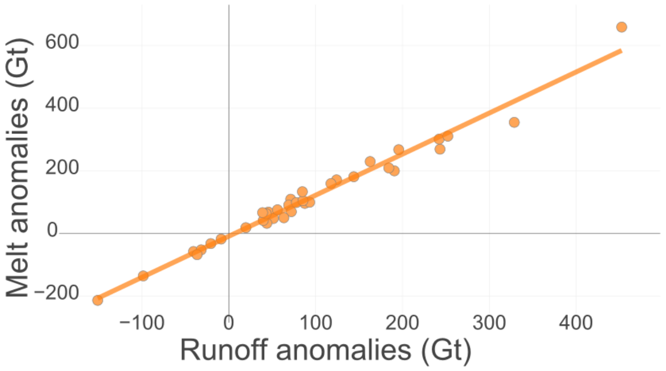

fig. S5. Correlation between annual melt and runoff anomalies. Scatterplot of annual melt anomalies against annual runoff anomalies, based on the 1970-1995 average of MAR ($R^2 = 0.98$, $p < 0.001$).
